# The Integration of Realistic Episodic Memories Relies on Different Working Memory Processes: Evidence from Virtual Navigation

**DOI:** 10.3389/fpsyg.2018.00047

**Published:** 2018-01-30

**Authors:** Gaën Plancher, Valérie Gyselinck, Pascale Piolino

**Affiliations:** ^1^Laboratoire Mémoire et Cognition, Université Paris Descartes, Paris, France; ^2^Institut de Psychologie, Université Paris Descartes, Boulogne Billancourt, France; ^3^Laboratoire d’Etude des Mécanismes Cognitifs, EA 3082, Université Lyon 2, Lyon, France; ^4^IFSTTAR-LPC, Versailles, France; ^5^INSERM U894, Centre de Psychiatrie et Neurosciences, Paris, France; ^6^Institut Universitaire de France, Paris, France

**Keywords:** virtual environment, episodic memory, working memory, binding, concurrent task

## Abstract

Memory is one of the most important cognitive functions in a person’s life as it is essential for recalling personal memories and performing many everyday tasks. Although a huge number of studies have been conducted in the field, only a few of them investigated memory in realistic situations, due to methodological issues. The various tools that have been developed using virtual environments (VEs) have gained popularity in cognitive psychology and neuropsychology because they enable to create naturalistic and controlled situations, and are thus particularly adapted to the study of episodic memory (EM), for which an ecological evaluation is of prime importance. EM is the conscious recollection of personal events combined with their phenomenological and spatiotemporal encoding contexts. Using an original paradigm in a VE, the objective of the present study was to characterize the construction of episodic memories. While the concept of working memory has become central in the understanding of a wide range of cognitive functions, its role in the integration of episodic memories has seldom been assessed in an ecological context. This experiment aimed at filling this gap by studying how EM is affected by concurrent tasks requiring working memory resources in a realistic situation. Participants navigated in a virtual town and had to memorize as many elements in their spatiotemporal context as they could. During learning, participants had either to perform a concurrent task meant to prevent maintenance through the phonological loop, or a task aimed at preventing maintenance through the visuospatial sketchpad, or no concurrent task. EM was assessed in a recall test performed after learning through various scores measuring the what, where and when of the memories. Results showed that, compared to the control condition with no concurrent task, the prevention of maintenance through the phonological loop had a deleterious impact only on the encoding of central elements. By contrast, the prevention of visuo-spatial maintenance interfered both with the encoding of the temporal context and with the binding. These results suggest that the integration of realistic episodic memories relies on different working memory processes that depend on the nature of the traces.

## Introduction

Early models of memory made clear distinctions between short-term and long-term memory. In 1890, James (1890) distinguished between primary and secondary memory. Primary memory, later renamed short-term memory, reflects current states of consciousness, while secondary memory, now referred to as long-term memory, consists of conscious memory of the past. This distinction was maintained in the majority of memory models (e.g., Waugh and Norman, 1965; Atkinson and Shiffrin, 1968). Since then, each construct has been investigated separately. This gave rise to many different theoretical models, mainly pertaining to the structuralist view. On the one hand, short-term memory evolved into the concept of working memory (WM), classically defined as a system dedicated to the temporary storage and the processing of information (Baddeley and Hitch, 1974). On the other hand, among several forms of long-term memory, the concept of episodic memory (EM) rapidly emerged. Episodic memories are typically described as long-term memories for which the mental experience includes specific information such as time, place, or perceptual details (Johnson and Raye, 1981; Tulving, 2002). Through a process of binding, the various items of information of EM, what-where-when, are linked together, forming connections that give a memory its specificity and distinctiveness (Johnson et al., 1993). Besides EM, different forms of long term memories exist. Semantic memory concerns the store of facts and general knowledge, including the mental lexicon. Implicit or non-declarative memory refers to a heterogeneous collection of non-conscious memory abilities including skills and habits, priming and simple conditioning (Squire, 1992).

While several scientific fields of research have led to a better understanding of these various forms of memory in the lab, the majority of studies seldom targeted realistic situations close to daily life, mainly due to methodological issues. Over the past decades, however, virtual environments (VEs) have gained popularity as a tool in cognitive psychology and neuropsychology because they enable researchers and clinicians to create naturalistic and controlled situations (e.g., for a review, Kane and Parsons, 2017; Plancher and Piolino, 2017). VEs can be developed for various situations. Depending on the study design, the environment can take the form of a city, an apartment, a store, a garden, etc. Interaction with the environment can be accomplished through a huge variety of devices, from a simple joystick or a keypad to a complex driving simulator. VEs have become a good candidate to study EM because they appear particularly suited to properly consider the various components of EM, for which an ecological evaluation is crucial.

Several factors have been identified as modulating the integration of episodic memories, e.g., organization learning (Roenker et al., 1971), level of processing (Craik and Lockhart, 1972), emotion (Kensinger and Corkin, 2003), etc. Some factors relate to the encoding stage, some to the consolidation stage, and others to the retrieval or recall stage. However, the interaction at encoding between WM and EM has rarely been directly assessed in naturalistic situations. This is particularly surprising, as the concept of WM has become central for understanding a wide range of cognitive functions. For example, WM capacities have been found to be involved in numerous areas of higher order cognition including language comprehension (Daneman and Carpenter, 1980; Gathercole and Baddeley, 1993), mathematics (Logie and Baddeley, 1987), reasoning (Engle et al., 1999), and spatial model construction (Gyselinck et al., 2007, 2009, 2015). As WM is connected with many cognitive functions, it is sometimes considered as the heart of cognition.

In several models, WM is seen as an interface between short-term perceptual memories and long-term memory, thus being of primary importance in the encoding process of future long-term memories. Models vary in their description of the way they interact, however (Ericsson and Kintsch, 1995; Cowan, 1999; Baddeley, 2000; Oberauer, 2002; Unsworth and Engle, 2007). Up to now, these models have mainly investigated the role of long-term memory in WM performance. In the present study, we aim rather at investigating the role of WM in the construction of each aspect of episodic traces, i.e., the traces of what, where and when.

The dual functions of storage and processing characterize WM functioning. Both processing and storage compete for attention, which is a limited resource. The WM model of Baddeley and Hitch (1974) distinguishes several components: the peripheral slave systems and the central executive system. The slave systems include the phonological loop which is necessary for the maintenance and the processing of verbal material, and the visuospatial sketchpad which is necessary for the maintenance and processing of visuospatial material. Finally, the central executive system manages the two slave systems (Baddeley and Hitch, 1974). Maintenance is of primary importance in most of the tasks and activities involving WM since, when both storage and processing are needed, participants usually tend as soon as possible to maintain the items to be remembered before processing them. Two mechanisms of maintenance have been distinguished in WM, articulatory rehearsal and refreshing (Baddeley et al., 1984; Barrouillet and Camos, 2012, respectively). Articulatory rehearsal has been described as being particularly involved in the maintenance of verbal material. The process of rehearsal can be blocked by articulatory suppression, i.e., a concurrent articulation of irrelevant verbal material (e.g., “babababa…”). Articulating this syllable involves a minimal cognitive load, but impairs memory performance of verbal information (Camos et al., 2009). The second maintenance mechanism is refreshing. It is primarily dedicated to visual and spatial material, even if the maintenance of verbal material can also rely on refreshing (Grillon et al., 2008; Camos et al., 2009). It enables the maintenance of memory traces through refocusing, i.e., thinking briefly of a just-activated spatial or visual representation.

In the present study, we investigate the role of WM in the construction of episodic memories using an original paradigm in a VE that enables all the components of EM (what, where, when, and binding) to be assessed. We address the question of whether preventing the verbal or visuospatial mechanism of maintenance in WM will have the same effect on the various EM traces of what, where and when. Although various methods have been developed to assess EM, few address entirely the original definition. Most of the time, EM is assessed with very simple tasks, e.g., remembering a word shown on a computer screen, which does not match the definition of EM as the ability to remember what, where, and when. Recently, some studies have begun to use a VE to assess episodic memories in ecological large-scale environments allowing a multi-component assessment of EM (Burgess et al., 2001; Sauzéon et al., 2011; Plancher and Piolino, 2017). In Plancher et al.’s studies, the usefulness of VEs has been demonstrated with young adults, healthy elderly and Alzheimer patients. Typically in these studies, participants were immersed in a VE in which they navigated via a video game wheel and followed a route composed of different turns. In addition to navigating, the participants were instructed to memorize all the elements of the scenes that they encountered within the environment, and to remember the temporal and spatial context associated with the elements so that they would be able to recall them at the end of the presentation. Some of the results suggested that assessing EM in a VE is more ecologic because the memory complaint was more highly correlated with the performances on the virtual test than with performances on the classical memory test (Plancher et al., 2012).

Some previous studies focused on the involvement of WM in spatial cognition using VEs. These studies can be considered as good assessments of the where component of EM. Meilinger et al. (2008) examined the WM involvement in a wayfinding task. Participants learned routes in a VE while they were disrupted by a visual, a spatial or a verbal secondary task. In the visual task, the participants had to imagine a clock with watch hands and indicate if the hands pointed to the same or different halves as the times they had heard. In the spatial task, the participants had to indicate where a sound was coming from (left, right, or front). In the verbal task, the participants had to perform a lexical-decision task. In all secondary tasks, participants received the stimuli via headphones and responded by pressing buttons on a response box. The authors observed that, compared to a control group, all secondary tasks interfered with wayfinding of the routes previously seen, by impacting the encoding of environmental information. The interference was stronger with the visual secondary task. According to the authors, the results indicate that the phonological loop and the visuospatial sketchpad are both involved in the encoding of environmental information. Meilinger et al. (2008) thus put forward a dual coding theory of human wayfinding.

In another virtual reality study, the involvement of WM in the construction of the mental representation of space was investigated (Gras et al., 2013). During route learning, the participants were asked to do a tapping task (tapping four keys sequentially in a parallelogram shape), or an articulatory suppression task (repeating “babebibobu”), or nothing, depending on the condition. Results showed different interference effects depending on the task (layout task vs. recognition of landmarks for example); in addition, the visuospatial abilities of WM modulated performance in the construction of the spatial model of a VE.

As far as we know, however, no experiment has yet assessed the role of WM by distinguishing the verbal and visuospatial subcomponents on different measures of episodic memories, that is, on EM in its entirety, i.e., what, where and when. In the present study, two secondary tasks were used. One focused on the verbal component, thus preventing the verbal rehearsal of episodic traces, while the other one focused on the spatial component, preventing the visuospatial refreshing of episodic traces. In the control condition, participants performed no secondary task.

The rationale of the present study is as follows. If an episodic trace (what, where, or when) relies on verbal maintenance and on the phonological loop, then the verbal secondary task performed during learning is expected to interfere with its encoding and hence result in a poorer recall. If an episodic trace relies on visuospatial representations requiring maintenance by refreshing, and the visuospatial sketchpad, the visuospatial secondary task should interfere also with its subsequent recall. More specifically, we assumed that factual traces (what) representing events and objects that could be easily verbalized should be maintained with verbal rehearsal. However, due to their visual nature they should also be maintained with refreshing. Thus, an interfering effect of both the verbal and the visuospatial concurrent tasks was expected. In contrast, the maintenance of spatio-temporal traces (where and when) and binding is probably less verbal and should be predominantly maintained with refreshing. Thus, mainly – if not only – interference with the visuospatial task was expected on performance reflecting the where, when and binding. The objective of the present study was to test these hypotheses in a more ecological paradigm than the ones traditionally used.

## Materials and Methods

### Participants

Eighty-eight undergraduate psychology students at the University (71 females, mean age = 20.32 years; *SD* = 1.71) received a partial course credit for participating. Each participant was randomly allocated to one of three groups (30 or 28 participants per group). We recorded the frequency with which participants played video games, and whether they had a driver’s license. Forty participants had a driver’s license and 51 participants regularly played video games. They were equally distributed over the three groups. However, to avoid an effect of familiarity with driving and video games on our results, before the presentation of the experimental environment, all the participants trained themselves on an empty track until they all felt comfortable with the apparatus. All participants gave their informed consent to the study, which was performed in compliance with the Declaration of Helsinki and with the approval of the University’s Institutional Review Board.

### Materials

#### The Virtual Equipment

The virtual equipment was composed of a computer-generated 3-D model of an artificial environment. This environment was built with Virtools Dev 3.0 software and the novel EditoMem and SimulMem softwares developed in the lab. The environment was run on a PC laptop computer and explored using a video-game steering wheel, a gas pedal, and a brake pedal. It was projected with a video projector onto a screen 85 cm high and 110 cm wide. The participants were seated in a comfortable chair. The VE was projected 150 cm in front of them.

#### The Virtual Environment

**FIGURE 1:**
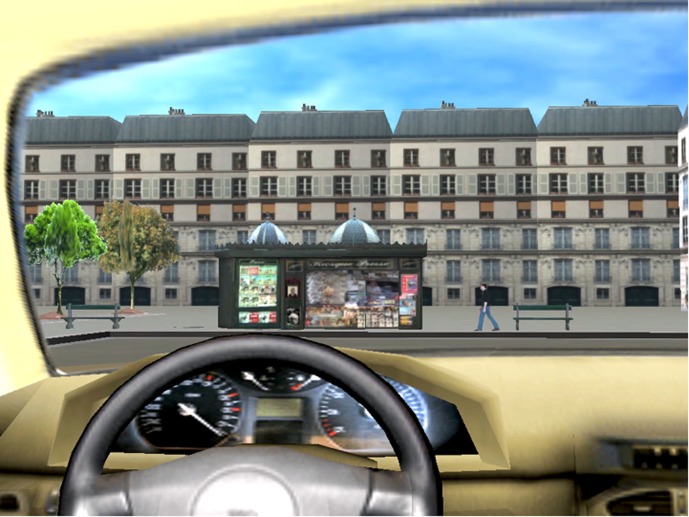
Picture of a specific area of the virtual town (a newsstand, a man and two benches).

An urban environment simulating French buildings was created. Since the participants were supposed to be sitting in a virtual car, the steering wheel and windshield were part of the images projected during the task (**Figure 1**). In the VE, one route connected ten specific scenes. Each specific scene comprised different elements: one central element (e.g., a newsstand or a sandwich shop) and two or three secondary elements (e.g., a man or a bench). The order in which the ten scenes were encountered (identified by the main element in each scene) was the following: a train station, a newsstand, a post office, a roadworks zone, a fountain, an old building, a parking lot, a sandwich shop, a car accident and a set of shops. Specific areas were located at a turn (**Figure 1**), and a soundtrack of typical city noises (cars, people, birds, etc.) heard through speakers helped the participants to feel immersed in the environment. No other vehicles were presented in the environment and no specific traffic rules had to be respected because, as presented on **Figure 1**, the spatial environment did not contain decision points (i.e., deciding to turn left or right).

**FIGURE 2:**
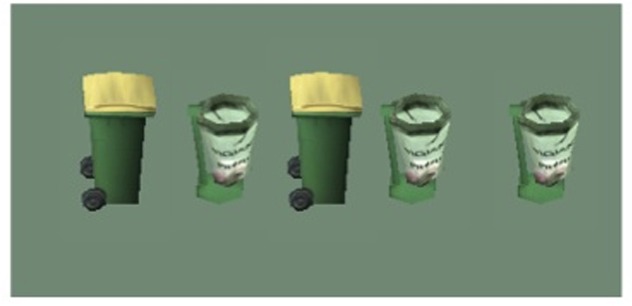
Example of a spatial arrangement of yellow and green garbage containers, all arranged along a straight line.

To be used for the secondary tasks, garbage containers were located on the sidewalks of the road. In the numerical secondary task assumed to interfere with the phonological loop the participants had to memorize the number of garbage containers. The containers were either green or yellow. The participants had to maintain the number of green and yellow garbage containers, respectively (there were six yellow and four green altogether). In the visuospatial secondary task, the participants had to memorize the spatial pattern composed by the garbage containers. They were displayed along a line in order to avoid the verbalization of visual forms such as “a square” or “a T.” They had to maintain the spatial arrangement of five containers (e.g., first position: yellow/second position: green/third position: yellow/fourth position: green/fifth position: green) (See **Figure 2**). A total of four patterns was used.

### Procedure

The condition of encoding was manipulated between-subjects. The same VE was used for all conditions. In all three conditions the participants were asked to drive into the town, without stopping, and to memorize all the elements of various scenes encountered in the town (what), along with the associated spatial locations (where) and the temporal context (when). An example scene not actually shown in the experiment was presented as a picture before the exploration, to ensure that the participants understood what they had to memorize: “If you encounter this scene in the virtual town, you have to memorize that there is a bakery, in the beginning of the town, and that this scene was located on a right-hand turn.”

Depending on the condition while driving in the virtual town the participants were disrupted by a secondary task that was either verbal or visuospatial. In the control condition, no secondary task was given. In the numerical condition, they were asked to memorize the total numbers of yellow and green garbage containers. Participants had thus to update the numbers each time a new garbage container was encountered. In the visuospatial condition they had to memorize the spatial arrangement of each pattern. The immersion ended when participants reached the edge of the town, which took around 3 min. Participants were informed that in the primary task, which involved the memorization of all the elements of the town, it was not necessary to include the garbage containers. Participants were instructed that both tasks, the primary and the secondary, were of equal importance.

Immediately after the immersion, the participants performed a recall test that assessed their performance in the secondary task. The participants of the verbal condition had to recall the total number of green and of yellow garbage containers. Participants of the visuospatial condition had to draw on a blank sheet the specific patterns in which the green and yellow containers were arranged. All recall tasks took 3 min. During this time, the participants of the control condition chatted with the experimenter.

After this first recall, we evaluated the participant’s performance in the EM test. In this test, we used a series of memory tests previously applied to assess EM with the same kind of paradigm (Plancher et al., 2010, 2012, 2013). Participants were required to perform a written free recall of all the elements they remembered, and when they remembered an element, they had to spell out the associated spatiotemporal context. The instructions were associated with an example as follows (each dependent variable is in brackets, associated with the maximum score):

- “Try to remember all the elements you saw in the town” (e.g., a grocery store, the restaurants) (number of *what* correctly recalled; max = 10)
- “Situate the elements in time: were they at the beginning, the middle, or at the end of the town?” (number of *when* correctly recalled; max = 10)
- “Try to remember if you turned left or right after the element” (number of *where* correctly recalled; max = 10).

The experimenter noted all recalls on a structured grid of responses. We did not take into account the recall of secondary elements (e.g., bench, tree, person) because they are too generic in a town and thus did not reflect the EM, we focused only on central elements (e.g., newsstand, train station, etc.). There was no specific order in the recall of the components. Once the element had been recalled, the participants could then provide contextual recall in any order. In total, 5 min were allowed for the recall.

In addition, we computed a binding score. For each element recalled, we noted whether the participants recalled the associated components (when and/or where). For example, if they recalled “the post office,” did they recall where and when it was presented (max by item = 2)? The binding score for a subject was the sum of all the contextual recalls (number of bindings correctly recalled; max = 20).

Performance in the secondary tasks was expressed as a percentage of correct responses (with 100% for all participants performing the control condition).

Prior to the beginning of the experiment, all participants underwent a training session in an empty environment (i.e., only streets) with a different spatial layout from that of the town subsequently used for the test. They were free to navigate anywhere on the training track. This training session provided the participants with an initial experience of a VE, and familiarized them with control of the virtual car. This session lasted until participants felt familiar with the equipment (on average around 4 min). After the training, the participants were immersed in the VE. The entire experiment lasted around 25 min, including the instructions.

We made the following hypotheses: while the number of *what* recalls should decrease with both secondary tasks, the number of *where*, *when* and *binding* should only decrease with the visuospatial secondary task.

## Results

Analyses were performed on the recall of each EM score (*what, when, where* and *binding*) through a series of ANCOVAs with the condition (verbal, visuospatial, no secondary task) as a between-subjects factor and the performance in the secondary task as a controlled variable. We decided to control this performance in order to avoid any influence of the task difficulty; the performance was expressed as a percentage of correct responses (with 100% for all participants performing the control condition). To determine the direction of the differences, we carried out *post hoc* Tukey tests. The following Tukey comparisons were analyzed: condition 1 (control) versus 2 (verbal secondary task) and condition 1 versus 3 (visuospatial secondary task). When the verbal or visuospatial secondary task conditions led to a poorer performance than the control condition, this was interpreted as reflecting an involvement of this component in the memorization of the episodic score. When both secondary tasks statistically differed from the control one, we performed the following Tukey comparison: condition 2 (verbal) versus condition 3 (visuospatial).

**FIGURE 3:**
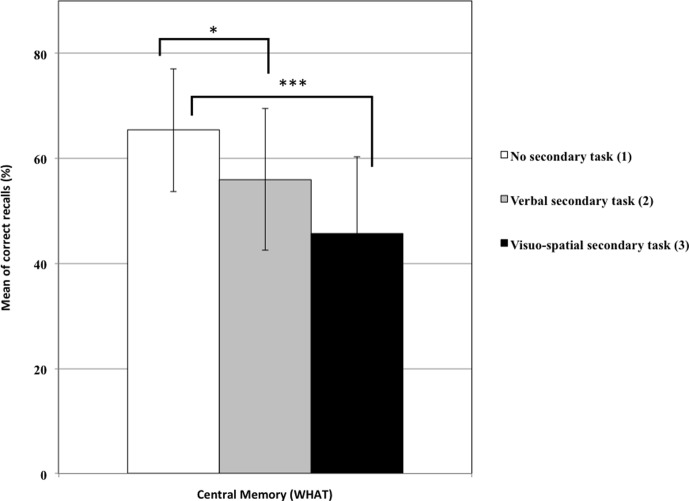
Percentage of central memory for the three experimental conditions (with standard deviation). Conditions 2 and 3 led to less recall than condition 1 (1 versus 2, ^∗^*p* < 0.05; 1 versus 3, ^∗∗∗^*p* < 0.001).

**FIGURE 4:**
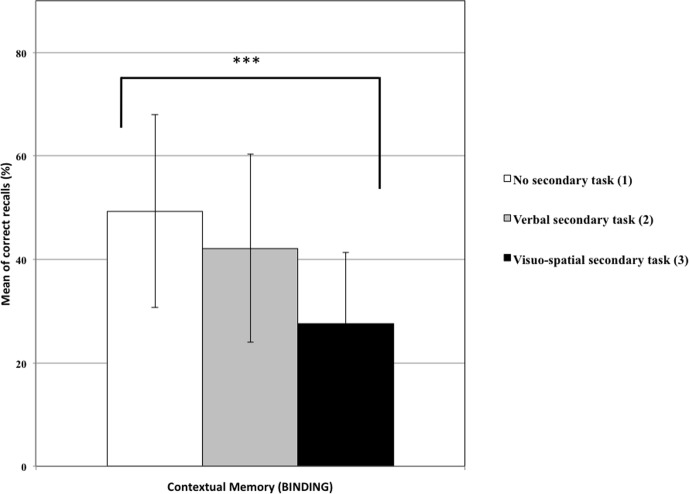
Percentage of contextual memory (binding) for the three experimental conditions (with standard deviation). Condition 3 led to fewer bindings than condition 1 (^∗∗∗^*p* < 0.001).

**Table 1** shows correct recall of the EM components with means and standard deviations by condition and the results of ANCOVAs and *post hoc* Tukey tests. A main effect of condition on the *What* recall was observed: as expected, participants performed better in the control condition (1) than in the other two (2 and 3) (**Figure 3**). This result suggests that memory traces related to central information can be maintained through verbal rehearsal or refreshing. Similarly, a significant effect of condition on the *When* recall was observed, but as expected only with the “visuospatial” condition (3) giving worse results than the control condition (1), suggesting that the temporal context is maintained through refreshing. Contrary to our predictions, no significant effect was observed on the *Where* score, which suggests that this score did not rely on WM maintenance. Finally, an effect of condition on *Binding* indicated that the “visuospatial” condition (3) gave worse results than the control condition (See **Figure 4**).

**Table 1:** Means and standard deviations of episodic scores for the various experimental conditions and results of ANCOVAs and *post hoc* Tukey tests.^1^

Score	No secondary task (1) *N* = 28	Verbal secondary task (2) *N* = 30	Visuo-spatial secondary task (3) *N* = 30	ANCOVA	*Post hoc* Tukey
What	6.54 (1.17)	5.60 (1.57)	4.57 (1.45)	*F*(2,84) = 6.09,	2 < 1, *p* = 0.04; 3 < 1,
				*p* = 0.003	*p* = 0.0001; 3 < 2, *p* = 0.02
When	4.86 (1.78)	4.13 (2.13)	2.70 (1.39)	*F*(2,84) = 3.85,	3 < 1, *p* = 0.0001
				*p* = 0.03	3 < 2, *p* = 0.008
Where	5.00 (2.64)	4.30 (2.65)	2.80 (1.81)	*F*(2,84) = 1.37,	
				*p* = 0.26.	
Binding	9.86 (3.73)	8.43 (4.11)	5.5 (2.8)	*F*(2,84) = 3.09,	3 < 1, *p* = 0.0001
				*p* = 0.049	3 < 2, *p* = 0.006

## Discussion

In daily life, we are continuously tasked with a long list of cognitive demands that must often be performed simultaneously. Often this means storing long-term memories while performing short-term tasks. Our aim in this experiment was to use a VE in order to test the role of WM while encoding episodic long-term memories in a naturalistic context. In particular, we tried to determine which component of WM is involved in the encoding of EM traces – distinguishing what, where, when and binding. Three main findings arose from our results. First, we observed that the memory of central information (what) was impaired by both numerical and visuospatial concurrent tasks. Second, the memory of temporal context and binding was impaired only when a visuo-spatial concurrent task was performed. Third, the spatial contextual recall was not influenced by any concurrent task.

Based on the assumption that the concurrent verbal and visuo-spatial tasks we used prevent, respectively, mainly rehearsal and refreshing, then our results indicate that central information is likely maintained by both verbal rehearsal and refreshing, whereas temporal and binding information are mainly maintained by refreshing. According to Baddeley’s model, the phonological loop is involved in the maintenance of verbal information (Baddeley, 1986). In most of the classical studies investigating the phonological loop, the material to be remembered in the primary task was letters or isolated words, items that are clearly verbal. In our study, the central information concerns objects, buildings and events encountered in our virtual city, which could be either reactivated in WM as images or as words. These items could be easily named (e.g., a train station, a post-office, etc.) and thus maintained through verbal rehearsal. Participants were instructed to intentionally memorize items encountered in the virtual town, as well as their context. It is thus likely that participants verbally rehearsed as soon as they could the name of items previously seen in order to avoid the traces decaying. We also observed that memorization of the central information was negatively affected when participants performed the visuospatial secondary task. As central items were presented visually, it is not surprising that the memory of central items also relied on visuospatial maintenance. This is consistent with the studies demonstrating that both maintenance mechanisms (verbal rehearsal and refreshing) can be run in parallel (e.g., Camos et al., 2009).

In addition, it is interesting to observe that the encoding of contextual long-term memories does not seem to rely heavily on verbal rehearsal, since reducing verbal rehearsal through a verbal memory task did not influence the memory context performance. This was true even when participants were instructed to encode the context. They could have developed a verbal strategy of maintenance (e.g., the newsstand was on my left when I turned), but apparently they did not. It seems that verbal strategies are not useful in the consolidation of contextual memories. Given that only the visuospatial secondary task prevented the encoding of contextual memory, refreshing seems to be the predominant mechanism of maintenance in that case. It is likely that verbal maintenance of contextual information is too costly and that maintaining different scenes through mental imagery is a more efficient strategy.

The memory of temporal and spatiotemporal binding information appears to be impaired only by visuospatial maintenance but the memory of the spatial component itself was not influenced by the visuospatial secondary task. This component was assessed by asking the participants to remember if they turned left or right after the element they recalled. This spatial recall is an egocentric one given that participants probably called upon their own body to answer. Egocentric processes are known to be viewpoint dependent and egocentric locations are updated by self-motion information (Burgess, 2006), and even across imagined self-motion (e.g., Burgess et al., 2004). In the present study, the visuospatial secondary task appeared rather to be allocentric in that it required participants to call upon external elements of the environment to encode the positions of the garbage containers. This would explain why our spatial secondary task did not interfere with our primary task. This interpretation is consistent with the findings of Farrell and Thomson (1998) and Farrell and Robertson (2000) which suggest that spatial egocentric information is automatically encoded through displacement in the VE and does not require to be maintained in WM. However, the high standard deviation of the control group may explain why statistical differences between groups difficulty emerged. This result should therefore be interpreted with caution.

Nevertheless, spatiotemporal binding information was negatively affected by the concurrent visuospatial maintenance. Loaiza and McCabe (2012) showed that refreshing is important for content-context binding in WM, and observed that the more refreshing opportunities an item receives, the more likely it is to be recalled from EM. These results are consistent with our findings suggesting that refreshing promotes memory of context and binding of EM traces. In the study by Loaiza and McCabe (2012), the context associated with central information was temporal in nature. They concluded that an item would be more stably bound to a temporal context when it is refreshed. In addition, previous work demonstrated that the memory of the temporal order in WM was maintained by using spatial mechanisms (e.g., Guida and Lavielle-Guida, 2014). For example, it seems that items to-be-remembered presented sequentially in the center of a screen acquired a spatial dimension: the first words of the sequence has a left spatial value while the last words has a right spatial value (van Dijck and Fias, 2011). Our present findings, which suggest that encoding of temporal memories was disrupted by a visuo-spatial concurrent task, are in accordance with these studies.

Our study presents some limitations, and these should be taken into account for future studies. In order to extend our knowledge of the mechanisms of maintenance involved in EM construction, in future work we could prevent and force rehearsal and refreshing more systematically. For example, we could continuously prevent verbal rehearsal by using an articulatory suppression (say “babababa”) or we could prevent attentional refreshing with a continuous auditory detection task. In that way we could separate the primary from the secondary task. In the present study, the primary and the secondary tasks involved both items presented in the VE. We cannot exclude the possibility that participants combined the two tasks. In addition, because the recall for the secondary task was performed before the primary task, it could have an influence on the recall of interest. In the future it would be important for the secondary task not to involve items of the primary task. In addition, to further improve the ecological validity of our assessment, the participants of the EM investigation should not receive any explicit instructions to memorize the episodic information (Pause et al., 2013). Finally, it could also be relevant to assess to what extent the degree of interaction between the VE and the participants, using higher immersive virtual navigation and virtual embodiment (Kilteni et al., 2012), mediates the encoding mechanisms of EM. It is conceivable that a greater immersion would give a stronger EM. Also, in our paradigm, participants drove a virtual car, which constituted a third task. It would be interesting to compare our results when participants perform passive navigation in the VE. The negative impact of the concurrent task could be reduced in that condition.

## Conclusion

Using an original paradigm of memory, our results demonstrate for the first time that preventing verbal maintenance through a concurrent task negatively impacts long-term memory of central information, while preventing visuospatial maintenance decreases central, temporal, and binding memory. WM thus appears central to consolidate EM reflecting everyday life and this maintenance is suggested to occur predominantly through the episodic buffer. Finally, as already demonstrated in long-term memory (Plancher et al., 2008, 2010, 2012, 2013), the ecological feature of a paradigm developed using VEs provides an excellent opportunity for investigating EM in its complexity.

## Ethics Statement

This study was carried out in accordance with the recommendations of APA, with written informed consent from all subjects. All subjects gave written informed consent in accordance with the Declaration of Helsinki. The protocol was approved by the committee of Paris Descartes University.

## Author Contributions

Substantial contributions to the conception or design of the work; or the acquisition, analysis, or interpretation of data for the work: GP, VG, and PP. Drafted the work or revised it critically for important intellectual content: GP, VG, and PP. Final approval of the version to be published: GP, VG, and PP. Agreement to be accountable for all aspects of the work in ensuring that questions related to the accuracy or integrity of any part of the work are appropriately investigated and resolved: GP, VG, and PP.

## Conflict of Interest Statement

The authors declare that the research was conducted in the absence of any commercial or financial relationships that could be construed as a potential conflict of interest.

## Footnotes

1 Results of the covariate: for what, when, where, and binding all *F* were <1.
